# Unraveling the role of rare earth dopants (Ce, La, Sm, and Y) in modifying the catalytic effect of TiO_2_ for MgH_2_ dehydrogenation: a first-principles analysis

**DOI:** 10.1039/d6ra05335a

**Published:** 2026-09-02

**Authors:** Xinru Zhu, Yaru Liu, Zhaohui Huang, Lumin Wang, Ling Wang, Min Li, Zhen Li, Yuexing Zhang, Longqing Yang, Xiaoli Wang

**Affiliations:** a Shandong Provincial Key Laboratory of Monocrystalline Silicon Semiconductor Materials and Technology, College of Chemistry and Chemical Engineering, Dezhou University Dezhou 253023 PR China wang52@mail.ustc.edu.cn; b Belgorod College of Food Sciences, Dezhou University Dezhou 253023 PR China; c School of Energy and Mechanical Engineering, Dezhou University Dezhou 253023 PR China; d Key Laboratory of Green Chemistry & Technology, Ministry of Education, College of Chemistry, Sichuan University Chengdu 610064 PR China

## Abstract

Magnesium hydride (MgH_2_) suffers from sluggish dehydrogenation kinetics owing to its strong Mg–H bonding, which limits its practical application. In this work, we present a systematic density functional theory (DFT) and climbing-image nudged elastic band (CI-NEB) study on the adsorption and dissociation behavior of MgH_2_ on rare-earth (RE = Ce, La, Sm, Y) doped TiO_2_(001) surfaces, with particular focus on the regulatory effects of doping configuration (substitutional *vs.* interstitial) and RE identity. Both doping modes are found to enhance surface charge transfer capability by introducing localized impurity states and narrowing the band gap. Beyond the conventional Mg–O charge transfer pathway, an additional RE–H coupling channel is identified, which plays a crucial role in stabilizing the adsorption configuration and weakening the Mg–H bonds, although its contribution varies with doping mode and RE element. More interestingly, the catalytic behavior differs markedly across dopants: on substitutionally doped surfaces, Ce, La, and Sm promote molecular adsorption of MgH_2_, whereas Y induces spontaneous and complete dissociative adsorption. On interstitially doped surfaces, Y and Sm lead to semi-dissociative adsorption of MgH_2_. Taking Ce doping as a representative case, the dehydrogenation energy barrier is substantially reduced from 0.48 eV on pristine TiO_2_ to approximately 0.13–0.15 eV. This study elucidates the atomic-scale catalytic enhancement mechanism of RE-doped TiO_2_ and provides a new perspective for the rational design of high-efficiency, low-cost RE-based hydrogen storage catalysts.

## Abstract 2

Beyond the Mg–O pathway, RE–H coupling stabilizes adsorption, weakens Mg–H bonds, and lowers the dehydrogenation barrier from 0.48 eV to 0.13 eV.

## Graphical abstract

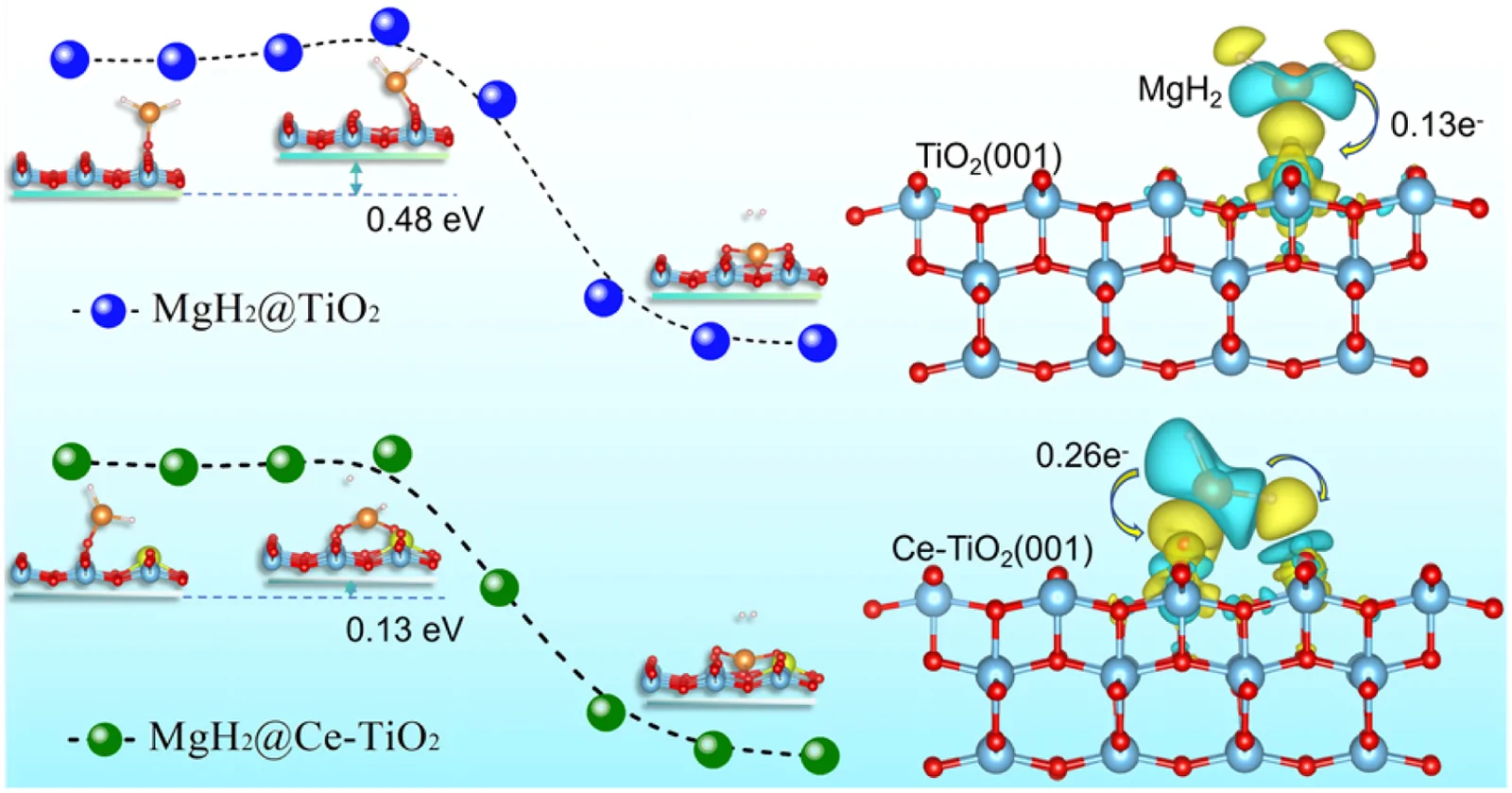

## Introduction

Hydrogen energy is widely regarded as a promising clean energy carrier owing to its high energy density and zero-carbon emission profile. However, the development of efficient hydrogen storage technologies remains a core challenge for its large-scale application.^1–3^ Among the various candidates, magnesium hydride (MgH_2_) stands out as a particularly attractive storage material due to its high gravimetric capacity, the relative abundance of magnesium, low cost, and good reversibility.^4–8^ Nevertheless, the strong Mg–H bonding results in unfavorable thermodynamics for hydrogen release, sluggish adsorption kinetics, and poor cycling stability.^9,10^ Therefore, developing effective strategies to alleviate these thermodynamic and kinetic limitations is of paramount importance, with catalyst design playing a pivotal role in this endeavor.

Transition metal-based catalysts have demonstrated significant potential in tuning the MgH_2_ dehydrogenation kinetics. Among them, Ti-based materials (*e.g.*, TiH_2_, TiCl_3_, and Ti-based intermetallics) have been extensively studied as effective catalytic additives owing to their distinctive electronic structures.^11–14^ As a representative candidate, TiO_2_ offers practical advantages, including high chemical stability and low cost. Nevertheless, the intrinsic wide band gap restricts electron mobility,^15,16^ while insufficient surface active sites limit catalytic H_2_ dissociation and recombination efficiency.^17^ To overcome these limitations, doping strategies have been employed to tailor the electronic structure of TiO_2_. For instance, Ti^3+^ self-doping (Ti^3+^@TiO_2_) promotes the generation of multivalent titanium species (Ti^3+^/Ti^4+^) *via* oxygen vacancies, which significantly lowers the hydrogen desorption temperature and MgH_2_ dehydrogenation activation energy.^18^ Similarly, the incorporation of Ni-doped TiO_2_ as a catalyst enhances the hydrogen storage kinetics of MgH_2_, enabling rapid hydrogen absorption at low temperatures and reducing the dehydrogenation activation energy by 40%.^19^ The observed kinetic enhancements are primarily attributed to the introduction of impurity energy levels,^20^ which modulate the band structure of TiO_2_, thereby promoting electron transfer and enhancing surface reactivity.

Rare earth (RE) elements offer unique advantages in modulating MgH_2_ dehydrogenation kinetics, which arise from their distinctive 4f electronic configuration.^21,22^ Doping with RE elements introduces highly active impurity states into the MgH_2_ band gap, effectively weakening Mg–H bonds and significantly lowering the dehydrogenation energy barrier.^23^ Nevertheless, direct doping of RE elements into the MgH_2_ matrix presents significant challenges, including agglomeration, the formation of stable rare-earth hydrides, and potential charge compensation effects that can suppress hydrogen diffusion. An alternative and more effective approach is to incorporate RE elements on solid catalyst surfaces.^24–26^ In this configuration, RE species introduce impurity levels that modify the catalyst electronic band structure, thereby enhancing its intrinsic catalytic activity. Specifically, the incorporation of RE ions in TiO_2_ has been shown to narrow the band gap through the formation of 4f–d hybridized states, promoting electron excitation and charge transfer at the catalyst surface.^27–29^ Despite these promising findings, the atomistic-level catalytic mechanism of RE-TiO_2_/MgH_2_ composites remains poorly understood. More critically, a systematic comparison of different RE dopants is still lacking, although previous studies have suggested that RE doping exerts distinct effects on the structure and catalytic performance of oxide supports.^30,31^

In this work, we have performed a systematic density functional theory (DFT) and climbing-image nudged elastic band (CI-NEB) study to elucidate the catalytic mechanism of RE-doped TiO_2_ (RE = Ce, La, Sm, and Y) for MgH_2_ dehydrogenation. These four elements were selected for their distinct 4f electronic configurations and ionic radii, with Y serving as a non-lanthanide reference to isolate the contribution of 4f electrons. A MgH_2_@RE/TiO_2_(001) interface model was constructed to analyze the interfacial electronic structure modifications and the resulting weakening of Mg–H bonds *via* density of states (DOS) and charge density difference analyses, while CI-NEB calculations were employed to identify transition states and determine the dehydrogenation energy barriers. The findings establish the atomistic origin of the catalytic enhancement observed for RE-doped TiO_2_ and provide a theoretical foundation for designing high-performance RE-based hydrogen storage catalysts.

## Model and method

### Calculation models

**Fig. 1:**
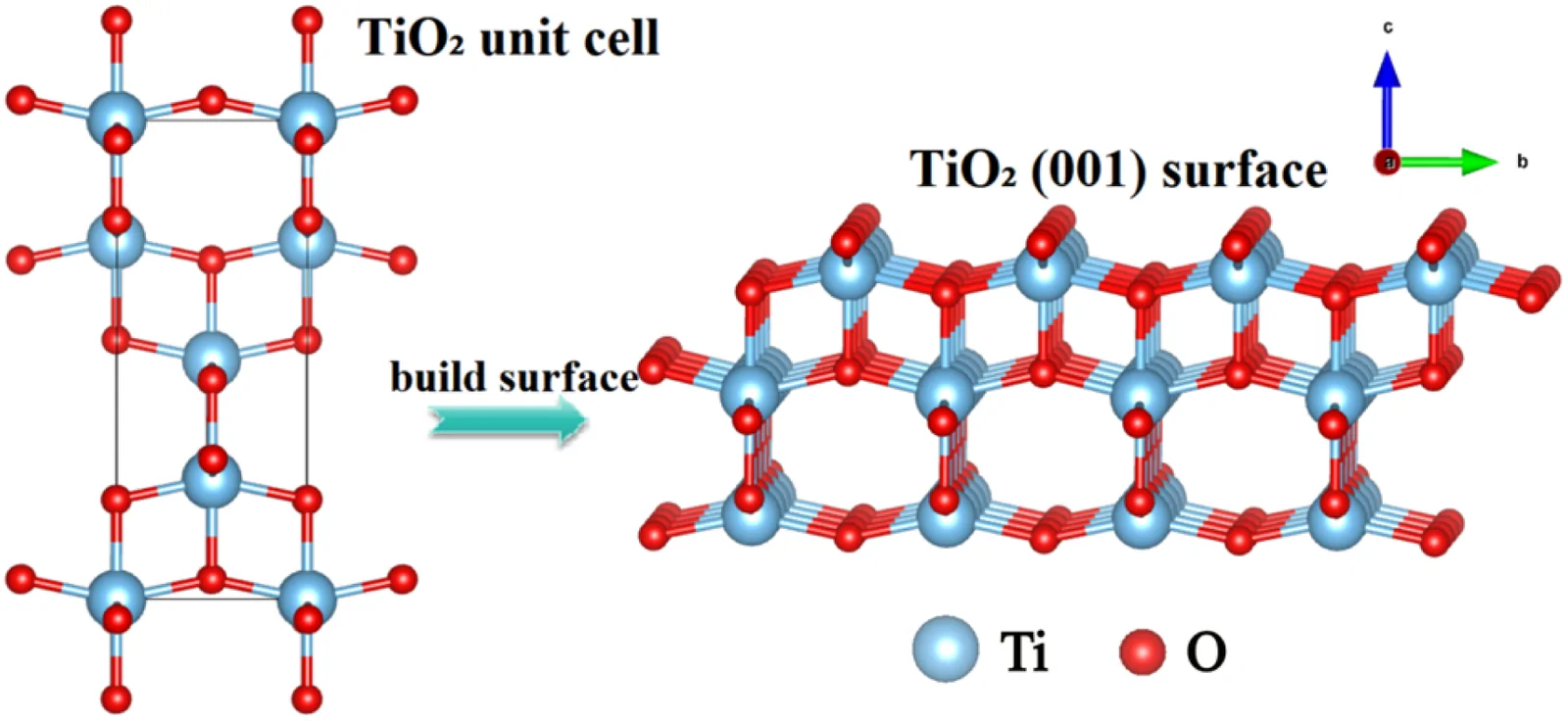
Structure of the anatase TiO_2_ unit cell and the supercell of TiO_2_(001) surface.

Tetragonal anatase TiO_2_ was employed as the substrate material. The primitive unit cell contains 4 Ti and 8 O atoms, with lattice parameters of *a* = *b* = 3.776 Å and *c* = 9.486 Å. A periodic slab model with a thickness of ≈6.2 Å was constructed by cleaving the bulk crystal along the (001) plane. A 4 × 3 supercell expansion in the *X* and *Y* directions was applied to generate a surface model containing 48 Ti and 96 O atoms (Fig. 1). Rare earth (RE = Ce, La, Sm, and Y) doping was achieved by either substituting the Ti lattice site or placing the RE atom at interstitial site within the TiO_2_ surface, yielding a doping concentration of ∼2%. To probe the catalytic interface, a single MgH_2_ molecule was placed on top of an oxygen atom closest to the dopant. The use of a single MgH_2_ molecule enables more precise determination of the energy barrier and electronic structure changes associated with the rate-limiting step, avoiding possible interference from intermolecular interactions in the condensed phase.

### Calculation methods

First-principles calculations were performed within the framework of DFT,^32,33^ as implemented in the Vienna *Ab initio* Simulation Package (VASP).^34,35^ The projector-augmented wave (PAW) method was utilized to express the interactions between ionic cores and valence electrons.^36,37^ The exchange–correlation energy was treated using the Perdew–Burke–Ernzerhof (PBE) generalized gradient approximation (GGA) functional.^38,39^ To account for the strong on-site Coulomb interactions of the 4f electrons, a Hubbard + *U* correction^40,41^ of 4.5 eV was applied to the rare-earth atoms, with this value chosen based on benchmark DFT + *U* studies on RE-doped oxide systems.^31,42,43^ A uniform U value was used for all RE dopants (Ce, La, Sm, and Y) to ensure internal comparability across different doping systems. A plane-wave basis set with a kinetic energy cutoff of 500 eV was employed. Structural relaxations were performed, applying periodic boundary conditions with a vacuum layer of 15 Å introduced along the *z* direction to avoid interactions between periodic images. The Brillouin zone was sampled using a 2 × 2 × 1 Monkhorst–Pack *k*-point grid. The convergence criteria were set at 10^−5^ eV for the total energy and 0.01 eV Å^−1^ for the maximum atomic force. The DFT-D3 method was employed to account for van der Waals dispersion interactions.^44^ The CI-NEB method^45,46^ was used to locate the transition states, for which six images inserted along the reaction pathway and a force convergence threshold of 0.05 eV Å^−1^.

## Results and discussions

### Rare-earth doping sites on TiO_2_

**Fig. 2:**
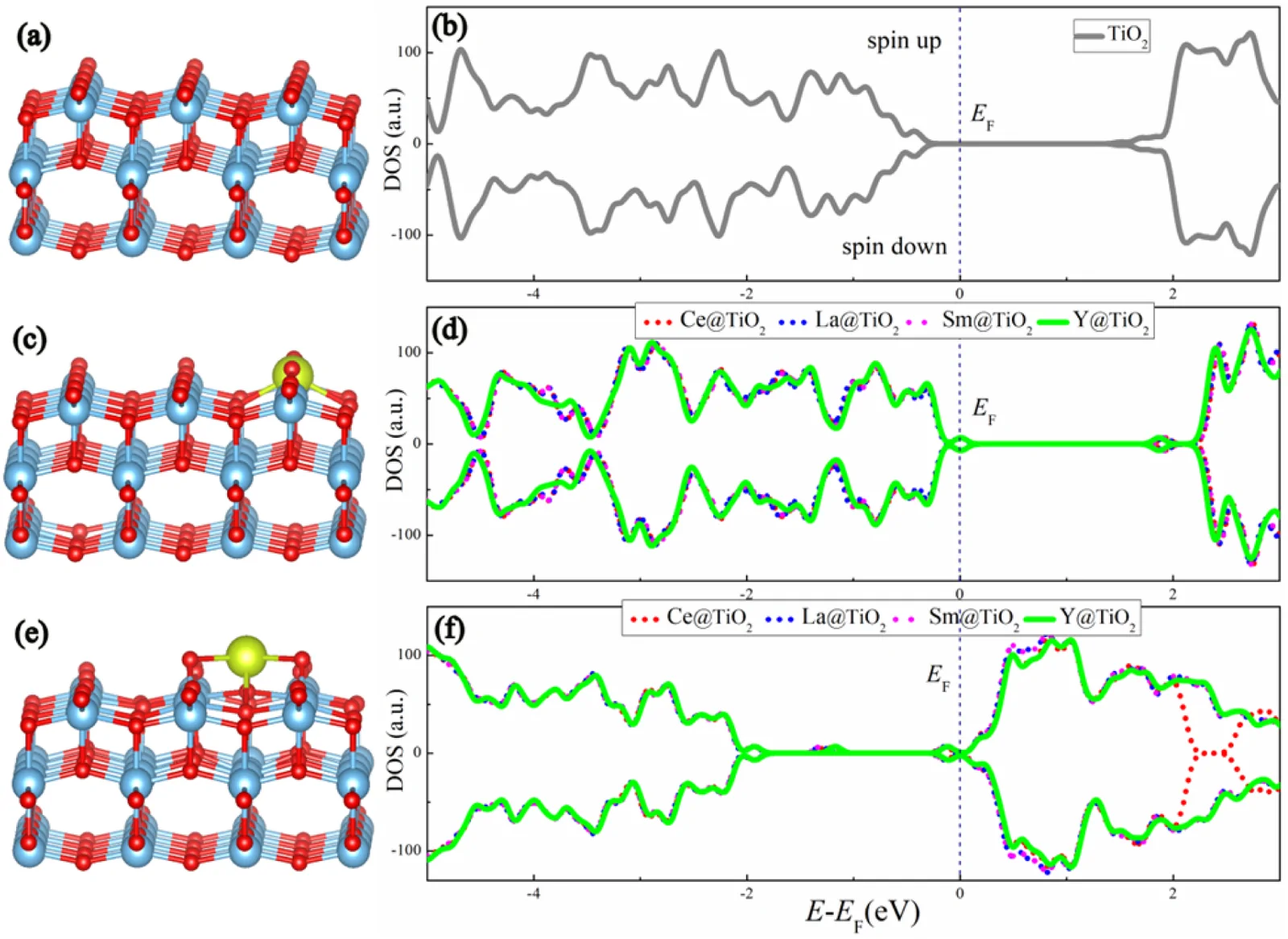
Atomic structures of (a) the pristine surface, (c) RE-doped surface *via* substitution, and (e) RE-doped surface *via* interstitial incorporation; density of states (DOS) for (b) the pristine surface, (d) RE-doped surface *via* substitution, and (f) RE-doped surface *via* interstitial incorporation.

On the pristine TiO_2_ surface, the surface Ti atom is five-fold coordinated with oxygen atoms (Fig. 2(a)). When a surface Ti atom is replaced by a rare earth (RE) element, the resulting configuration remains stable and the RE dopant also adopts five-fold oxygen coordination (Fig. 2(c)), while the system remains non-magnetic. In contrast, RE doping at interstitial sites within the surface layer (Fig. 2(e)) introduces local structural distortion and alters the coordination environment of the RE dopant to four surface oxygen atoms. Notably, this interstitial configuration induces a finite net magnetic moment of 0.83–0.85 *µ*_B_, which is predominantly localized on the Ti atom adjacent to the RE dopant, see Sec. S5 of the (SI).

The electronic structure of the TiO_2_ surface can be significantly modulated by the choice of doping methodology. As shown in Fig. 2(b), the pristine TiO_2_ surface exhibits semiconducting behavior, characterized by a calculated band gap of approximately 1.88 eV and the Fermi level (*E*_F_) located within the gap, which gives rise to a negligible density of states at *E*_F_. Consequently, this electronic configuration leads to pronounced chemical inertness and the absence of effective surface active sites.

Substitutional doping with RE atoms (Ce, La, and Sm) induces a significant reconstruction of the electronic structure, as illustrated in Fig. 2(d). Specifically, the Fermi level shifts downward to the valence band maximum (VBM), while the DOS is redistributed accordingly. The band gap narrows from 1.88 eV to 1.69 eV, accompanied by the presence of DOS near the Fermi level. The Y-modified TiO_2_ surface displays a DOS distribution with less overlap compared to the former cases, and its band gap is further narrowed to 1.65 eV.

In the case of interstitial doping, DOS analysis reveals a downward shift of the occupied states, resulting in a shift of the *E*_F_ toward the conduction band minimum (CBM). This shift can be attributed to the donor-doping behavior of Ce, in which electrons are transferred from the dopant to TiO_2_, thereby raising *E*_F_. Across all RE-doped (Ce, La, Sm, and Y) system, no significant differences are observed in the DOS distributions near the Fermi level. The band gap of the interstitial-doped systems is slightly narrowed to 1.59 eV compared to the pristine TiO_2_ surface. The localized DOS emerging within the band gap arises from the interaction between the surface states and the impurity states induced by rare-earth atoms, see Fig. S2 in the SI.

### Adsorption structure of MgH_2_ on the surface

**Fig. 3:**
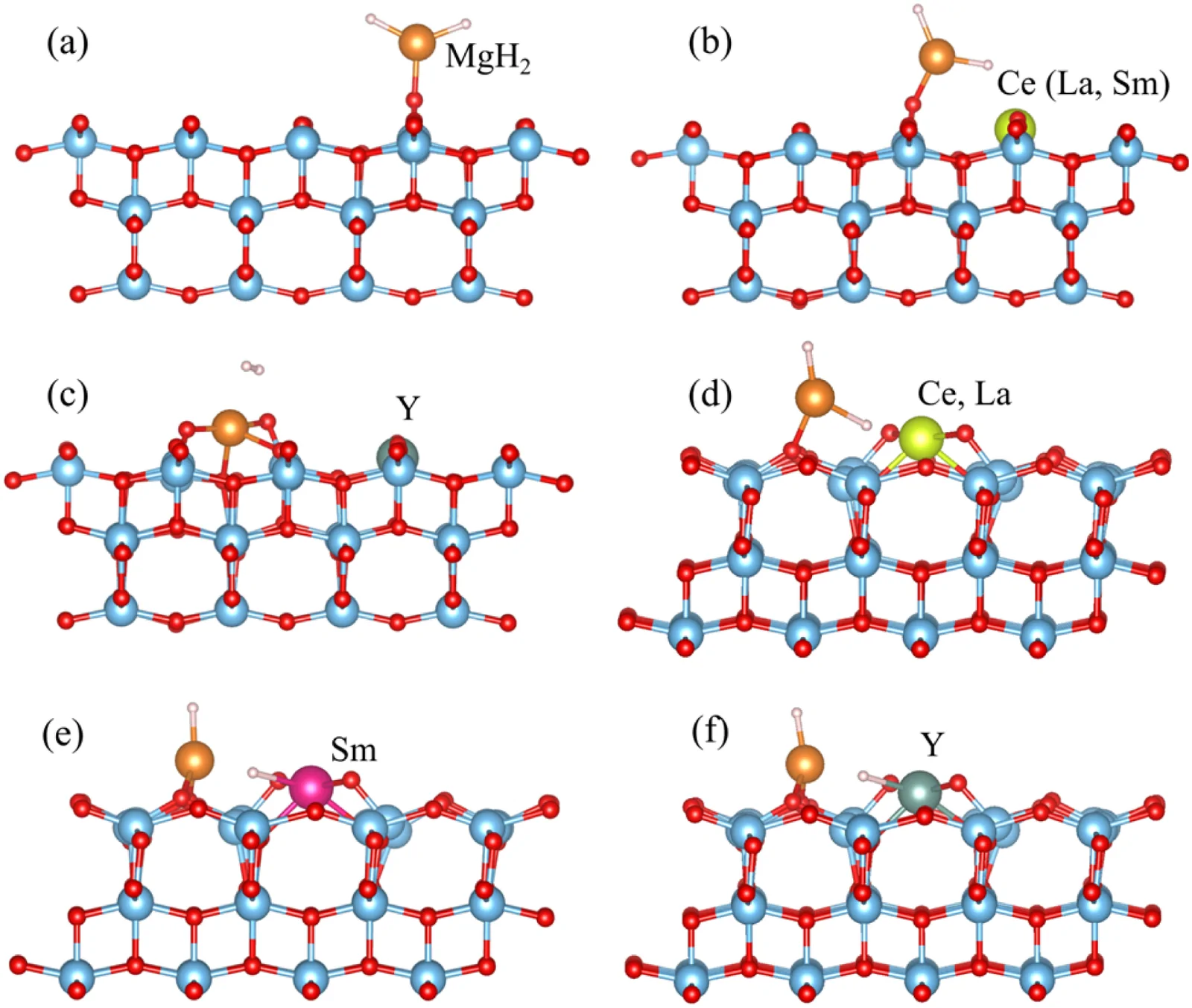
Side views of the optimized MgH_2_ adsorption configurations on TiO_2_(001) surfaces: (a) undoped; (b) substitutional RE-doped (RE = Ce, La, Sm); (c) substitutional Y-doped; (d) interstitial Ce (La)-doped. (e) Interstitial Sm-doped; (f) interstitial Y-doped.

The optimized MgH_2_ adsorption configuration on the unmodified TiO_2_(001) surface is shown in Fig. 3(a). In this configuration, the H–Mg–H bond angle is approximately 132°, with both H atoms tilted upward. The Mg atom coordinates to the top site of a surface oxygen atom, forming a chemical bond. The calculated bond lengths are 1.76 Å for the two equivalent Mg–H bonds and 1.96 Å for the Mg–O bond. The Mg–O bond length is notably shorter than that associated with crystalline MgO (∼2.10 Å),^40^ consistent with a strongly polar covalent character. The surface oxygen atom is significantly displaced outward from its ideal lattice position, which is mainly attributed to the hybridization between the O p-orbitals and the Mg s-orbital (see Fig. S3 of the SI).

The adsorption energy was estimated from:

1 *E*_ads_ = *E*_tot_ − *E*_sub_ − *E*_MgH_2__

where *E*_tot_, *E*_sub_ and *E*_MgH_2__ represent the energy of the composite system, the corresponding substrate (pristine or doped), and an isolated MgH_2_ molecule, respectively. A negative value of *E*_ads_ corresponds to a stable and exothermic adsorption configuration. For the undoped TiO_2_ surface, the computed *E*_ads_ is −3.53 eV, confirming that the adsorption process is highly exothermic and thus thermodynamically favorable.

Ce substitution for a Ti atom induces significant local structural distortion of the TiO_2_ surface. Although the initial adsorption site remains the same as on the undoped surface, the final optimized configuration exhibits marked changes. As shown in Fig. 3(b), the MgH_2_ molecule tilts axially toward the Ce atom, bringing one H atom closer to the surface, while the Mg–H bond length is slightly elongated. Correspondingly, the adsorption energy becomes more negative (−3.8 eV). Moreover, in addition to the strong coupling between the Mg s-orbital and O p-orbitals, additional hybridization also occurs between the Ce atom and one of the H atoms (see Fig. S4 in the SI).

**Fig. 4:**
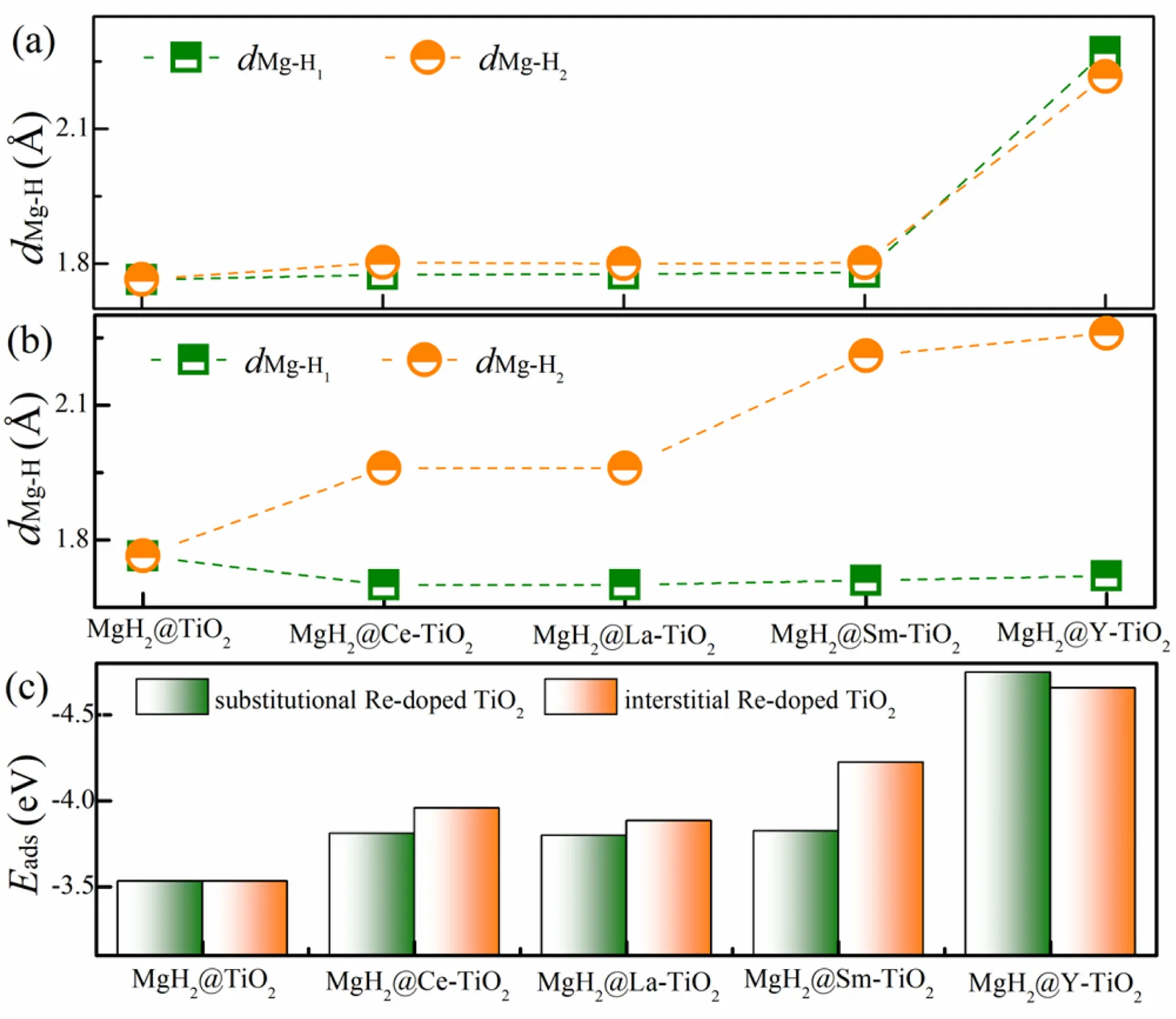
(a) Comparison of Mg–H bond lengths on substitutionally doped TiO_2_ surfaces; (b) comparison of Mg–H bond lengths on interstitially doped TiO_2_ surfaces; (c) adsorption energies of MgH_2_ on the TiO_2_ surface under different RE doping conditions.

The effects induced by La and Sm doping are consistent with those observed for Ce doping. The geometric distortions (see Fig. S1 in SI), bond length variations, and adsorption energies exhibit excellent consistency, as illustrated in Fig. 4(a, c) and Table 1. In contrast to the Ce-, La- and Sm-doped systems, the Y-doped TiO_2_ surface exhibits a distinctly different MgH_2_ adsorption configuration (Fig. 3(c)). The Mg atom moves away from the Y dopant to a distance of 6.08 Å and coordinates with four surface oxygen atoms. The elongation of Mg–H bond length to 2.27 Å suggests MgH_2_ dissociation and the formation of an H_2_ molecule. The dissociative adsorption is accompanied by a significantly more negative adsorption energy (−4.74 eV), confirming a unique catalytic effect of Y doping in promoting MgH_2_ decomposition.

**Table 1:** Key structural parameters and adsorption characteristics for MgH_2_ adsorption on pristine and RE-doped TiO_2_(001) surfaces

Surface	Doping mode	*d*_Mg–H1_ (Å)	*d*_Mg–H1_ (Å)	*d*_Mg–O_ (Å)	*E*_ads_ (eV)	Adsorption type
Pristine	—	1.76	1.76	1.96	−3.53	Molecular
Ce-TiO_2_	Substitutional	1.78	1.80	1.91	−3.81	Molecular
La-TiO_2_	Substitutional	1.78	1.80	1.90	−3.80	Molecular
Sm-TiO_2_	Substitutional	1.78	1.80	1.91	−3.82	Molecular
Y-TiO_2_	Substitutional	2.27	2.22	2.14	−4.74	Dissociative
Ce-TiO_2_	Interstitial	1.7	1.96	1.92	−3.96	Molecular
La-TiO_2_	Interstitial	1.7	1.96	1.92	−3.89	Molecular
Sm-TiO_2_	Interstitial	1.71	2.21	1.99	−4.22	Semi-dissociative
Y-TiO_2_	Interstitial	1.72	2.26	2.00	−4.65	Semi-dissociative

The adsorption behavior of MgH_2_ on interstitially doped TiO_2_ surfaces differ from that on substitutionally doped systems, see Fig. 3(d–f). For the distal part of the molecule relative to the interface, the Mg–H bond length remains nearly unchanged; however, in the near-surface region, the bond length shows a strong dependence on the dopant. As illustrated in Fig. 4(b, c) and Table 1, Ce and La doping lengthen the near-surface Mg–H bond to 1.96 Å, accompanied by a significantly more negative adsorption energy. In the case of Sm-doped surfaces, the bond length is further extended to 2.21 Å with an adsorption energy of −4.22 eV, suggesting complete Mg–H bond dissociation. On Y-doped surface, the bond length reaches 2.26 Å with the most negative adsorption energy (−4.65 eV), indicating the strongest adsorption and dissociation capability among all interstitially doped systems.

Consequently, MgH_2_ adsorbs primarily in a molecular form on substitutionally doped TiO_2_ surfaces, with complete dissociative adsorption occurring only in the case of Y doping. In contrast, semi-dissociative adsorption is observed on interstitial-doped surfaces. The distinct catalytic response can be traced to subtle differences in electronic structure, as illustrated in Fig. 2. Specifically, the localized impurity states that emerge near the Fermi level can serve as electron reservoirs that inject charge into the Mg–H antibonding orbitals. This electron injection thereby directly weakens the Mg–H bonds and governs the varying degrees of adsorption observed across the different doping configurations.

### Charge transfer analysis of MgH_2_ on RE-doped TiO_2_

**Fig. 5:**
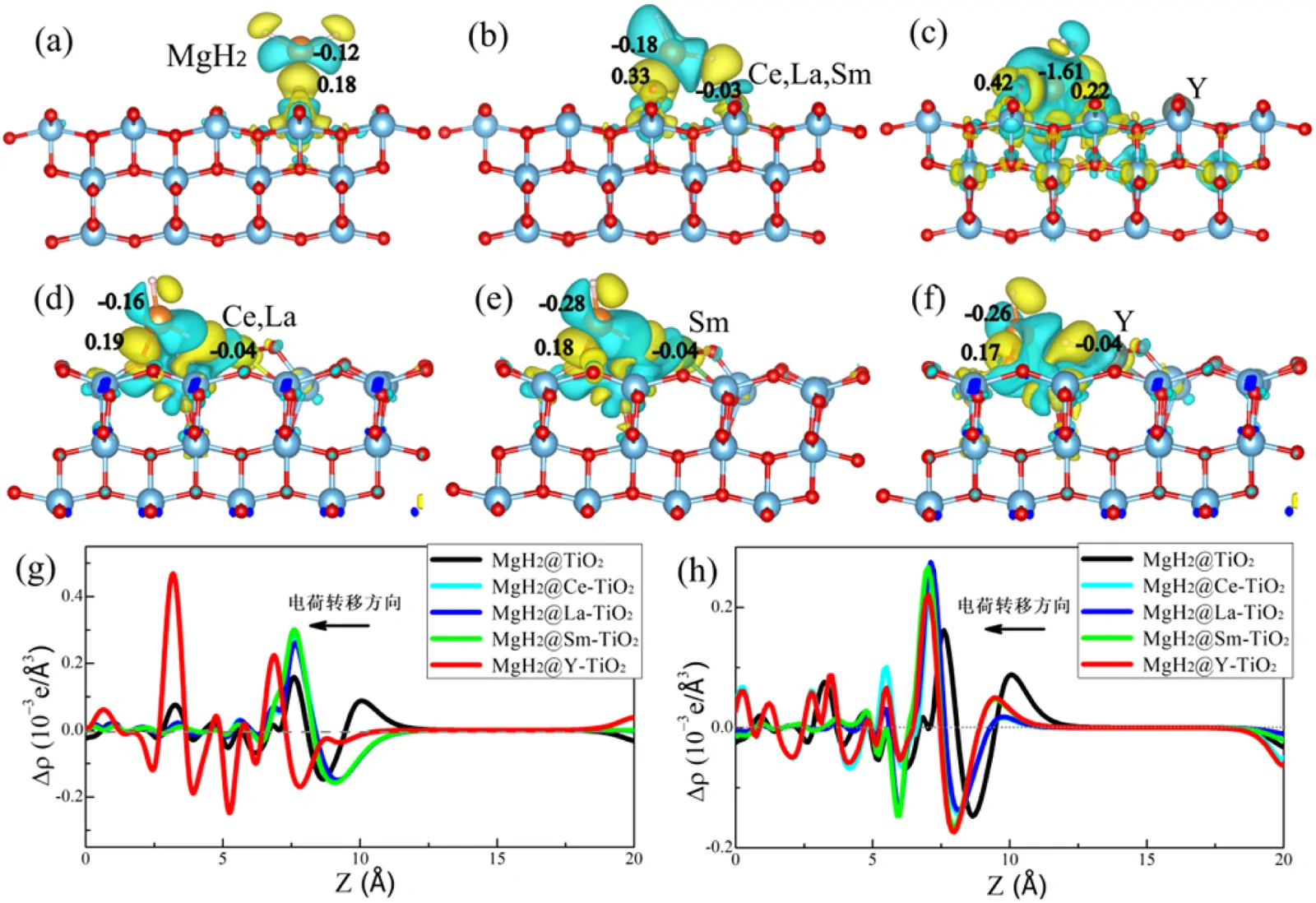
Charge density difference (Δ*ρ*, isosurface level = ±0.001 e per bohr^3^) for the MgH_2_ adsorption configuration on different TiO_2_ surfaces. (a) Pristine TiO_2_, (b) substitutional Ce (La, Sm)-doped surfaces, and (c) substitutional Y-doped surface; (d) interstitial Ce (La)-doped surfaces, (e) interstitial Sm-doped surface, and (f) interstitial Y-doped surface; in-plane averaged Δ*ρ* for (g) substitutionally doped and (h) interstitially doped systems.

The charge density difference, which reveals the direction and magnitude of charge transfer between the surface and the adsorbate, is given by:

2 Δ*ρ* = *ρ*_tot_ − *ρ*_sub_ − *ρ*_MgH_2__

where *ρ*_tot_, *ρ*_sub_ and *ρ*_MgH_2__ are the charge densities of the total system, the substrate, and the free MgH_2_ molecule, respectively. Charge accumulation (Δ*ρ* > 0) and depletion (Δ*ρ* < 0) are represented by yellow and green isosurfaces, respectively. On the pristine TiO_2_ surface, charge redistribution largely confined to the region around the Mg atom and the surface O atom to which it bonds, giving rise to Mg–O charge transfer channels, as evidenced by Fig. 5(a) and Bader charge analysis (Table S3, SI). Charge analysis reveals that the adsorption process is predominantly driven by strong electrostatic interactions: the Mg atom loses about 0.12 e^−^ and is indirectly coupled to a surface Ti^4+^ site *via* the bridging surface O atom, which gains about 0.18 e^−^, ultimately forming localized Mg–O–Ti bonding configurations.

The region of charge redistribution is significantly expanded for surfaces doped substitutionally with rare-earth elements (Ce, La, and Sm), as illustrated in Fig. 5(b). In addition to the Mg–O charge transfer channels, a notable electron density redistribution is observed between the doped RE atom and an H atom of MgH_2,_ as quantified by Bader charge analysis showing that the H atom gains 0.04 e^−^ and the RE atom loses 0.04 e^−^ (Table S3, SI). This indicates a considerable coupling between the RE atom and the H atom, which plays a crucial role in stabilizing the adsorption configuration. On the Y-doped TiO_2_ surface (Fig. 5(c)), the MgH_2_ moves away from the Y site after adsorption, preventing a direct charge transfer channel between Y and H. Charge redistribution becomes more extensive, focusing primarily on the Mg atom and its surrounding surface O atoms. Quantitatively, Bader charge analysis shows that Mg loses 1.61 e^−^ on the Y-doped surface, while the four surface O atoms coordinated to Mg each gain 0.22–0.42 e^−^, with no net charge transfer detected on Y. Notably, both the intensity and spatial range of the charge density difference are markedly enhanced in this configuration.

The in-plane averaged Δ*ρ* quantifies the interfacial charge accumulation and depletion upon adsorption. The positive and negative peaks shown in Fig. 5(g) correspond to regions of net charge accumulation and depletion, respectively. For the pristine TiO_2_ surface, a prominent positive-negative peak pair appears near *z* = 8.0 Å, which corresponds to a charge transfer of approximately 0.13 e^−^ across the Mg–O interface. This finding offers quantitative evidence for charge transfer from Mg to O. On the Ce-, La-, and Sm-substituted surfaces, the charge transfer associated with this peak pair increases to 0.26 e^−^. This suggesting that beyond the primary Mg–O charge transfer channel, additional charge transfer between the rare-earth element and the adjacent H atom further enhances this effect. In contrast, the profile obtained for the Y-doped surface shows more pronounced features: a significantly greater charge oscillation amplitude and a broader spatial range of charge redistribution. Moreover, the newly formed bonding interface between the Mg atom and four surface O atoms allows charge transfer to extend deeper into the substrate.

For the interstitial doped TiO_2_ surfaces, the charge density difference after MgH_2_ adsorption exhibits notable differences, as shown in Fig. 5(d) and (f). Compared to the pristine TiO_2_ surface, interstitial doping introduces an additional RE–H charge transfer channel. The degree of charge redistribution varies among the different doped systems, with the order: Y/Sm-doped surfaces >Ce/La-doped surfaces, as quantified by Bader charge analysis (Table S3, SI). The in-plane Δ*ρ* analysis (Fig. 5(h)) further reveals that the primary charge–transfer interface on the doped surfaces is shifted closer to the substrate, suggesting stronger coupling between the substrate and the adsorbed MgH_2_ molecule. The interfacial charge transfer derived from the positive-negative peak pairs amounts to 0.16 e^−^ (for Y), 0.15 e^−^ (for Sm), and 0.13 e^−^ (for Ce/La), respectively.

The impurity states introduced by doping narrow the band gap of the TiO_2_ surface (see Fig. 2), effectively lowering the energetic barrier for interfacial electron transfer and promoting charge redistribution between the surface and the adsorbate. Beyond this electronic structure modulation, RE–H coupling is commonly observed in RE-doped TiO_2_ systems, although its presence and strength depend strongly on the intrinsic electronic structure, ionic radius, and doping configuration of the specific RE element (see the discussion in sec. S2 of SI). On substitutionally doped Ce/La/Sm surfaces as well as interstitially doped configurations, the rare-earth impurities mediate adsorption through RE–H coupling and associated electron transfer. Notably, substitutional Y doping lacks direct Y–H interaction and instead promotes dissociative adsorption through global modulation of the Mg–O charge redistribution.

### Dissociation of MgH_2_ on the surface

**Fig. 6:**
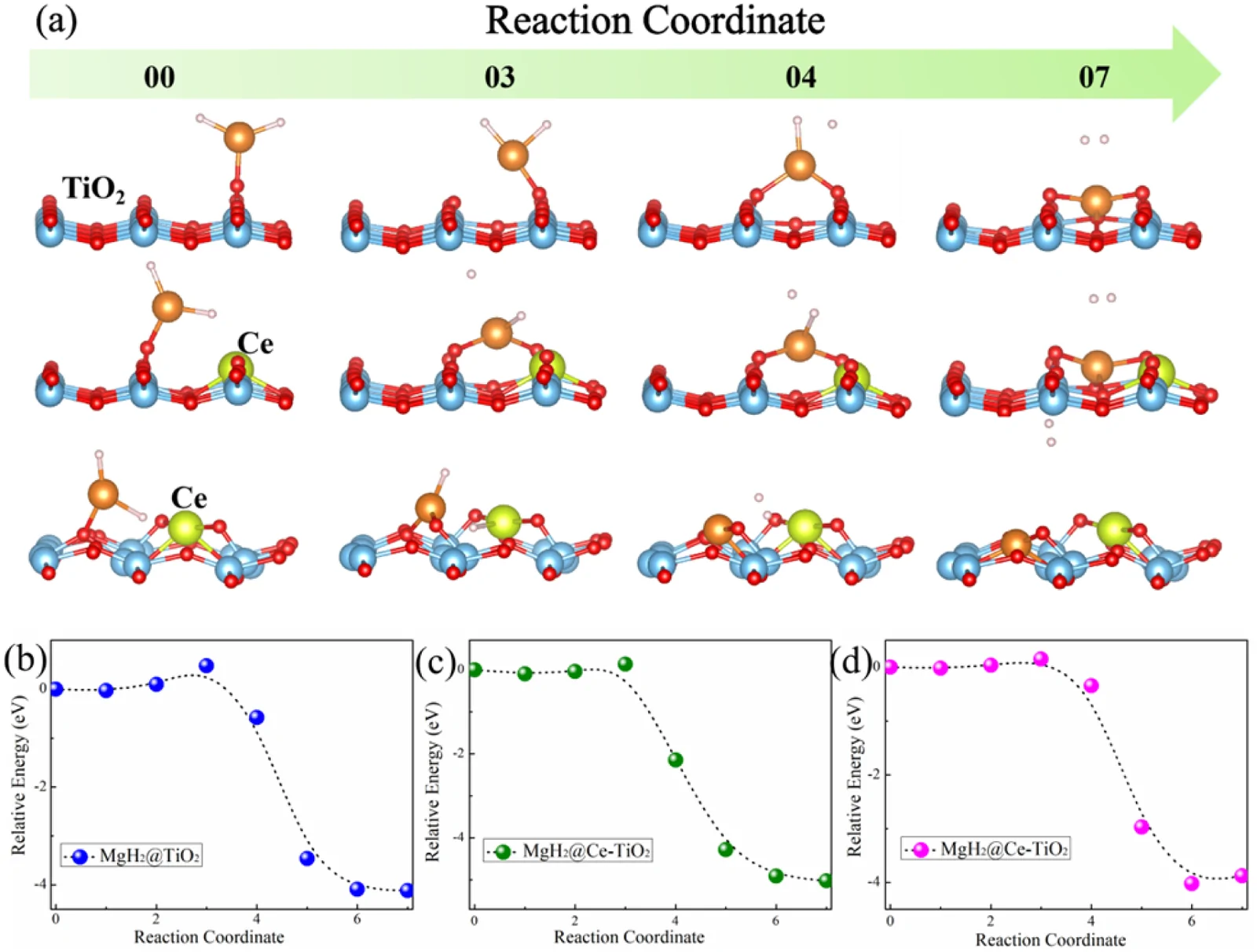
(a) Schematic illustration of H_2_ dissociation from an MgH_2_ molecule on pristine TiO_2_, Ce-substituted doped TiO_2_, and Ce-interstitial doped TiO_2_ surfaces. The relative energy profiles for the dissociation process on (b) pristine TiO_2_, (c) Ce-substituted doped TiO_2_, and (d) Ce-interstitial doped TiO_2_.

The above analysis confirms that the dissociative adsorption of MgH_2_ on the Y-doped surface is a thermodynamically spontaneous and barrierless process, which precludes the definition of a distinct transition state for substitutional Y doping. La and Sm substitutional doping, owing to their structural and energetic similarity to Ce, are expected to exhibit dehydrogenation barriers comparable to that of Ce. Ce thus serves as an ideal representative, capturing the general enhancement effect of RE doping while offering a well-defined reaction pathway and computational feasibility. The dissociation pathways and corresponding energy barriers are presented in Fig. 6.

The structural evolution during MgH_2_ dissociation on the three surfaces is illustrated in Fig. 6(a). For the pristine TiO_2_ surface (first row), the MgH_2_ molecule is initially adsorbed vertically atop a surface oxygen atom. As the reaction proceeds, the Mg atom tilts to coordinate with two surface oxygen atoms, accompanied by an elongation of the Mg–H bonds (see Sec. S4 in SI). In the final state, the Mg atom coordinates with four surface oxygen atoms, leading to the complete dissociation of MgH_2_ and recombination of the two released hydrogen atoms into an adsorbed H_2_ molecule.

For the Ce-substituted TiO_2_ surface (Fig. 6(a), second row), MgH_2_ initially adopts a tilted adsorption configuration. During the reaction, the Mg atom gradually coordinates with two surface oxygen atoms, which promotes the preferential dissociation of one hydrogen atom. Ultimately, the Mg atom coordinates with four oxygen atoms, and the remaining hydrogen atom subsequently dissociates to form an H_2_ molecule. By contrast, on the Ce-interstitial doped surface (Fig. 6(a), third row), the Ce dopant captures one of the hydrogen atoms, causing it to detach from the MgH_2_ molecule. Subsequently, as Mg coordinates with surface oxygen atoms, both H atoms dissociate and recombine to form an H_2_ molecule.

The transition state for the dehydrogenation on the pristine TiO_2_ surface exhibits a single imaginary frequency in the vibrational analysis, corresponding to the energy maximum along the reaction pathway, as illustrated in Fig. 6(b). This transition state is characterized by an elongated but not yet fully broken Mg–H bond. The calculated energy barrier for this process is 0.48 eV, comparable to the barrier reported for a V_2_O_5_ surface (0.6 eV)^47^ and significantly lower than the decomposition barrier of bulk MgH_2_ (>2 eV).^48^ This barrier suggests that although the reaction is thermodynamically favorable, it nevertheless requires a certain kinetic activation energy to proceed.

Compared with the pure TiO_2_ surface, Ce doping substantially enhances catalytic activity by markedly reducing the dehydrogenation energy barrier. As shown in Fig. 6(c) and (d), the dissociation of MgH_2_ on the Ce-TiO_2_ surface is highly exothermic, with the final-state energy lying considerably below the initial state. A less pronounced local energy maximum is observed at coordinate 03, which corresponds to the transition state. The calculated energy barriers for this process are 0.13 eV for substitutional doping surface and 0.15 eV for interstitial doping surface—both significantly lower than that obtained for the pristine TiO_2_ surface. These findings indicate that Ce doping effectively lowers the kinetic barrier, thereby facilitating MgH_2_ dehydrogenation and contributing to high catalytic efficiency.

In addition to the Ce-doped system, we further performed CI-NEB calculations for Y interstitial doping (see Sec. S7 of SI). The potential energy profile shows a monotonic decreasing trend after adsorption, suggesting either a spontaneous barrierless process or an extremely low barrier below our detection limit. In either case, the conclusion remains that Y doping effectively promotes MgH_2_ dehydrogenation.

## Conclusions

In this study, DFT calculations combined with the CI-NEB method are employed to systematically investigate the catalytic mechanism of rare-earth (RE = Ce, La, Sm, Y) doped TiO_2_ for the dehydrogenation of MgH_2_. By constructing substitutional and interstitial doping configurations on the TiO_2_(001) surface, we reveal that both doping modes narrow the band gap, introduce localized impurity states near the Fermi level, and enhance charge transfer capability. The adsorption configuration and dissociation degree of MgH_2_ are highly dependent on both the doping site and the specific RE element. Specifically, on substitutionally doped surfaces, Ce, La, and Sm promote molecular adsorption, whereas Y doping triggers spontaneous dissociative adsorption with a considerably more negative adsorption energy (−4.74 eV). In contrast, Y and Sm interstitial doping led to semi-dissociative adsorption. Charge density difference further uncovers an additional RE–H coupling channel beyond the primary Mg–O charge transfer pathway. Furthermore, CI-NEB calculations demonstrate that Ce doping markedly lowers the dehydrogenation energy barrier from 0.48 eV on pristine TiO_2_ to approximately 0.13–0.15 eV. Together, these results elucidate the atomic-scale origin of the catalytic enhancement effect of RE-doped TiO_2_ in MgH_2_ hydrogen storage systems, offering theoretical guidance for the rational design of high-performance, low-cost RE-based catalysts.

## Author contributions

Xinru Zhu: conceptualization (lead); writing – original draft (lead). Yaru Liu: methodology (lead); conceptualization (equal); Zhaohui Huang: visualization (lead). Lumin Wang: visualization (equal); writing – original draft (equal). Ling Wang: writing – original draft (equal). Min Li: software (equal). Zhen Li: writing – review and editing (equal). Yuexing Zhang: formal analysis (equal); methodology (equal). Longqing Yang: software (lead). Xiaoli Wang: formal analysis (lead); writing – review and editing (lead).

## Conflicts of interest

There are no conflicts to declare.

## Supplementary Material

RA-OLF-D6RA05335A-s001

## Data Availability

The authors confirm that all data supporting the findings of this study are provided within the paper and its supplementary information (SI) files. Further data can be obtained from the corresponding author upon reasonable request. Supplementary information: additional computational details and electronic structure analyses related to this study. See DOI: https://doi.org/10.1039/d6ra05335a.
