# Supplementary material for: Unraveling the role of rare earth dopants (Ce, La, Sm, and Y) in modifying the catalytic effect of TiO2 for MgH2 dehydrogenation: a first-principles analysis
Source: RSC Adv. 2026 Sep 2;16(43):52624–31. doi: 10.1039/d6ra05335a (PMC13537203; doi:10.1039/d6ra05335a)
Supplement: Supplementary file 1 [file RA-016-D6RA05335A-s001.pdf]

Supplementary Information (SI) for RSC Advances.
This journal is © The Royal Society of Chemistry 2026

Supplementary Information for

Unraveling the Role of Rare Earth Dopants (Ce, La, Sm, and Y) in Modifying the Catalytic Effect of TiO_2 for MgH_2 Dehydrogenation: A First-Principles Analysis

Xinru Zhu,[a] Yaru Liu,[b] Zhaohui Huang,[a] Lumin Wang,[a] Ling Wang,[a] Min Li,[c] Zhen Li,[a] Yuexing Zhang,[a] Longqing Yang[d] and Xiaoli Wang[a]*

AFFILIATIONS

[a] *Shandong Provincial Key Laboratory of Monocrystalline Silicon Semiconductor Materials and Technology, College of Chemistry and Chemical Engineering, Dezhou University, Dezhou 253023, PR China.*

[b] *Belgorod College of Food Sciences, Dezhou University, Dezhou 253023, PR China.*

[c] *School of Energy and Mechanical Engineering, Dezhou University, Dezhou 253023, PR China.*

[d] *Key Laboratory of Green Chemistry & Technology, Ministry of Education, College of Chemistry, Sichuan University, Chengdu 610064, PR China.*

*Corresponding author

E-mail: wang52@mail.ustc.edu.cn

CONTENT

S1. Convergence tests for computational parameters 3

S2. Distinct catalytic behavior of Y compared to Ce, La, and Sm 4

S3. DOS analysis for RE-TiO_2 surface and MgH_2 adsorption system 6

S4. Structural evolution during H_2 desorption on Ce-doped TiO_2 surfaces 9

S5. Spin distribution of RE interstitially doped TiO_2 11

S6. Bader charge analysis of the MgH_2 adsorption systems 12

S7. CI-NEB result for MgH_2 dehydrogenation on Y-doped TiO_2 surface 13

S1. Convergence tests for computational parameters

Prior to the systematic calculations, we performed convergence tests for both the plane-wave cutoff energy and the k-point mesh, as shown in Table S1. For the cutoff energy, we tested values of 400, 450, 500, 550, and 600 eV. The results show that the total energy differs by less than 1 meV/atom when increasing the cutoff from 500 to 550 eV, confirming that 500 eV is well converged. For the k-point mesh, given the large vacuum layer along the z-direction in our surface slab model, only one k-point is required in this direction. We tested 1×1×1, 2×2×1, 3×3×1, and 4×4×1 Monkhorst–Pack grids. The energy difference between 1×1×1 and 2×2×1 is only 0.2 meV/atom, and further increasing to 3×3×1 yields a negligible change (0.04 meV/atom). We therefore adopted a 2×2×1 mesh to ensure a sufficient safety margin for all calculations.

Table S1 Convergence of total energy with respect to cutoff energy (ENCUT) and k-point mesh.

Parameter	value	Energy (eV/arom)	ΔE (meV/atom)
ENCUT (eV)	400	-8.04781	--
	450	-8.03771	10.1
	500	-8.02644	11
	550	-8.0255	**0.94**
	600	-8.02573	0.23
K-point mesh	1*1*1	-8.02644	--
	2*2*1	-8.02665	0.2
	3*3*1	-8.02669	**0.04**
	4*4*1	-8.0267	0.01

S2. Distinct catalytic behavior of Y compared to Ce, La, and Sm

The adsorption configuration of the MgH_2 molecule is identical on the substitutionally Ce-, La-, and Sm-doped TiO_2 surfaces, where the molecule adopts a tilted geometry with an intact Mg–H bond. In contrast, on the Y-doped TiO_2 surface, a distinctly different configuration is observed (Fig. S1(a)), wherein the Mg–H bond is fully dissociated and the two H atoms recombine into an H_2 molecule adsorbed on the surface. On the interstitially doped TiO_2 surfaces, the MgH_2 adsorption configuration is similar for Ce and La doping, where one Mg–H bond is elongated but not broken (Fig. S1(b)). In contrast, for Sm and Y doping, one Mg–H bond is fully dissociated, and the detached H atom bonds with the rare-earth dopant.

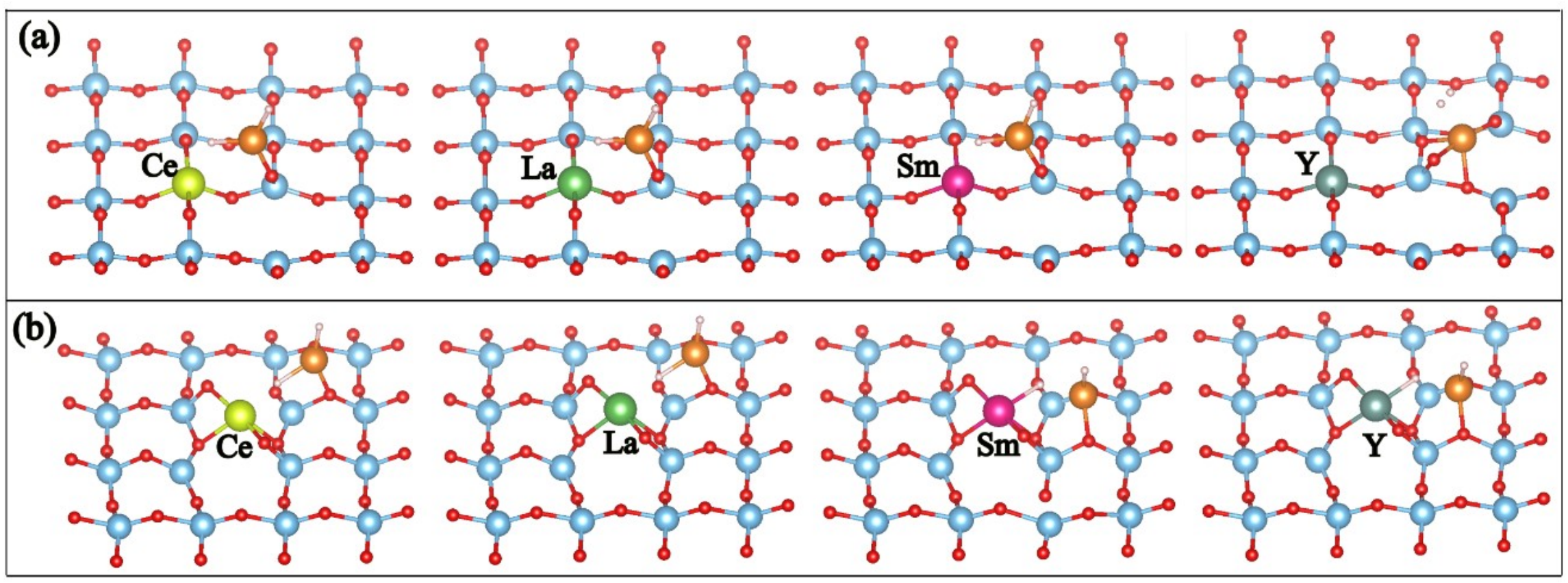

Fig. S1 Top view of MgH_2 molecule on (a) substitutional RE-doped (RE = Ce, La, Sm, Y) TiO_2 surface; (b) interstitial RE-doped TiO_2 surface.

To elucidate the electronic structure origin of the distinctly different catalytic behavior of Y compared to Ce, La, and Sm, we summarize the key electronic structure differences as follows.

1) Influence of 4f electronic configuration and doping configuration on RE–H coupling: Ce, La, and Sm possess partially filled or empty 4*f* orbitals that can hybridize with H 1*s* states, forming the RE-H coupling channel. In contrast, Y has an empty 4*f* shell and thus lacks this 4*f*-H hybridization capability. However, whether Y can form RE-H coupling depends strongly on the doping configuration. In substitutional doping, Y occupies a Ti lattice site at a relatively large distance from the adsorbate, where the spatial overlap between its 4*d* orbitals and H 1*s* states is limited, preventing effective Y-H hybridization. Consequently, substitutional Y doping drives dissociative adsorption through enhanced Mg-O charge redistribution. In interstitial doping, Y is stabilized at a surface interstitial site in closer proximity to the adsorbate, enhancing the spatial

overlap between 4*d* and H 1*s* orbitals and enabling Y-H coupling via 4*d*-H 1*s* hybridization, which results in semi-dissociative adsorption.

2) Influence of ionic radius and local structural stability: The ionic radius of Y is closer to Ti^{4+} compared to Sm, Ce, and La. This closer ionic radius match provides higher structural stability for Y in substitutional doping, facilitating its effective incorporation into Ti lattice sites.

In summary, the electronic structure, doping configuration, and ionic radius collectively determine the presence and strength of RE–H coupling, thereby governing the distinctly different catalytic behaviors observed.

S3. DOS analysis for RE-TiO_2 surface and MgH_2 adsorption system

On the interstitial-doped TiO_2 surface, rare-earth atoms introduce impurity states within the band gap (as shown in Fig. S2). These impurity states interact with the surface states, thereby inducing a spin-polarized DOS distribution near -1 eV.

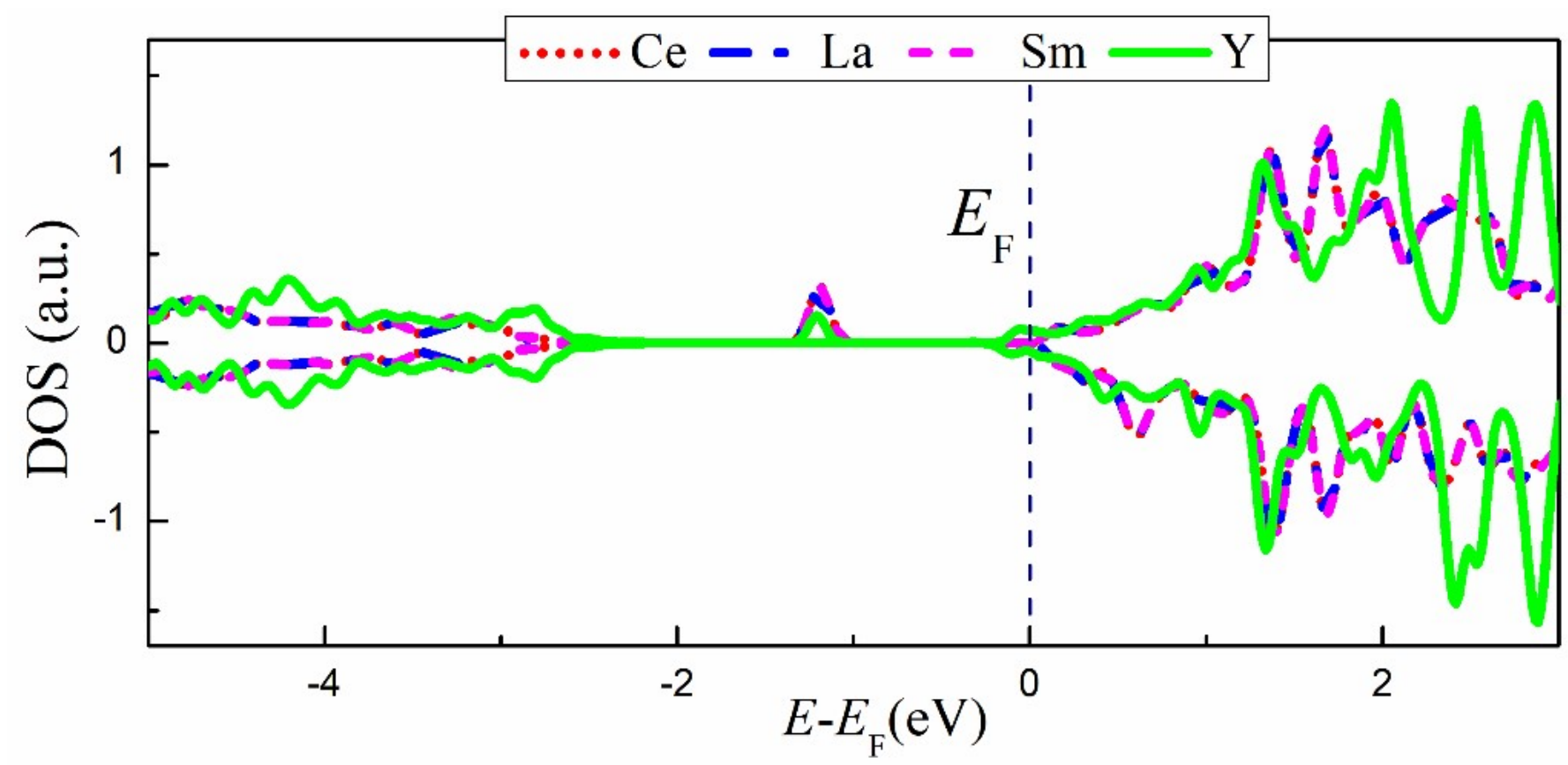

Fig. S2 DOS contribution of rare-earth atoms on the interstitial-doped TiO_2 surface

For the MgH_2 molecule adsorbed on the pristine TiO_2 surface, the DOS analysis is presented in Fig. S3. As shown in Fig. S3(a), the Ti atoms exhibit negligible DOS contribution near the Fermi level. In Fig. S3(c) and S3(d), both the Mg *p*-orbitals and the H *s*-orbitals display sharp peaks around the Fermi level, with a significant energy overlap, indicating pronounced hybridization between the Mg *p* and H *s* states. Furthermore, the O atom directly bonded to Mg exhibits density of states peaks at the energy position corresponding to the Mg *s* orbital and near the Fermi level (Fig. S3(b)), confirming the hybridization between Mg and O.

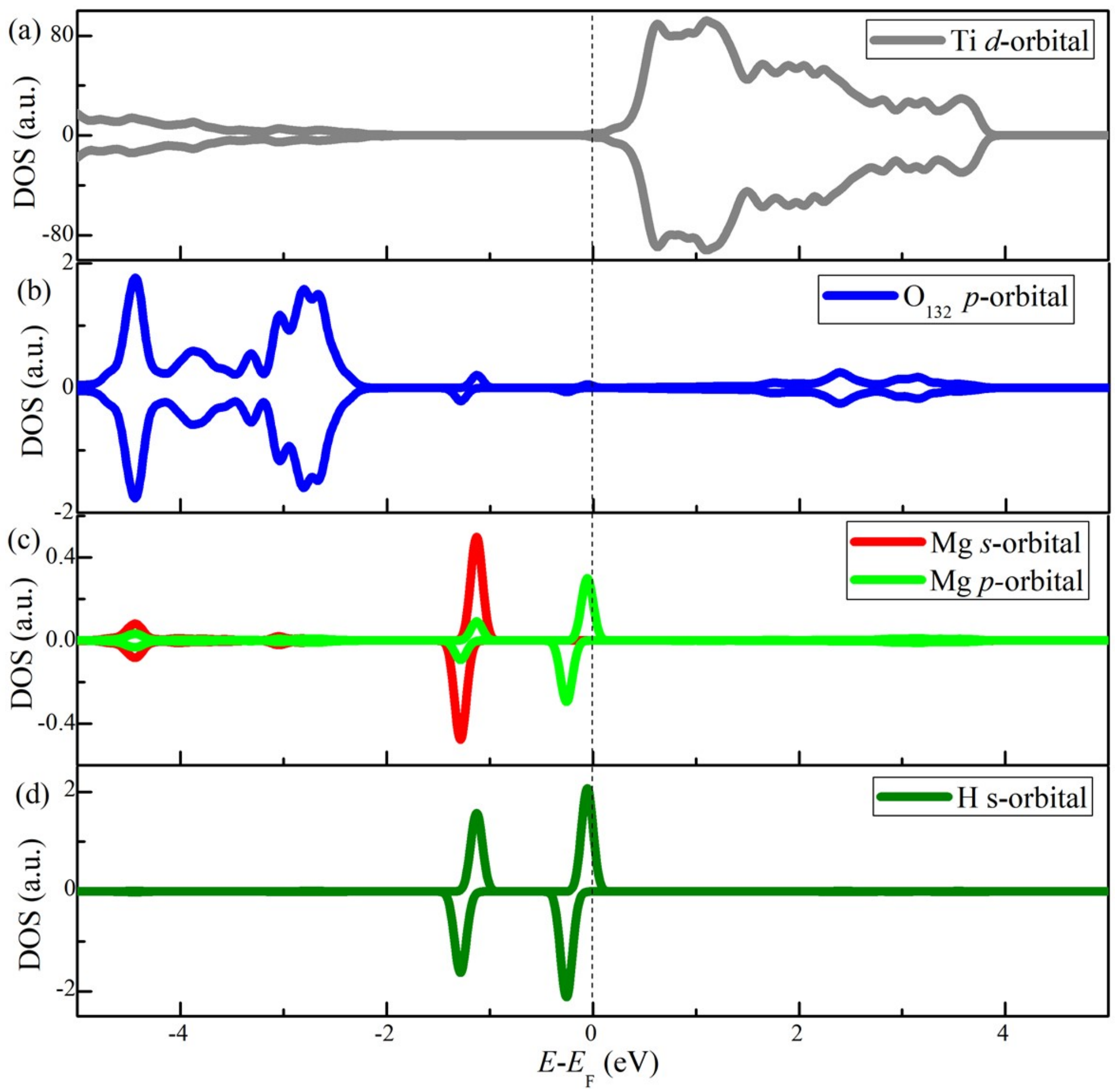

Fig. S3 The projected density of states (PDOS) of MgH_2 on TiO_2 surface ($E - E_F = 0$ eV) for (a) Ti *d*-orbitals, (b) O *p*-orbitals, (c) Mg *s*- and *p*-orbitals, and (d) H *s*-orbitals.

For the MgH_2 molecule adsorbed on the Ce-doped TiO_2 surface, the DOS analysis is presented in Fig. S4. As shown in Figs. S4(c) and S4(d), both the Mg *p*-orbitals and the H s-orbitals exhibit sharp peaks near the Fermi level, indicating that strong hybridization between the Mg *p* and H *s* states is maintained. Meanwhile, as displayed in Figs. S4(a) and S4(b), the Ce atom and the O atom directly bonded to Mg also show sharp peaks at the Fermi level, confirming the effective hybridization coupling between Ce and H, and between O and Mg, respectively.

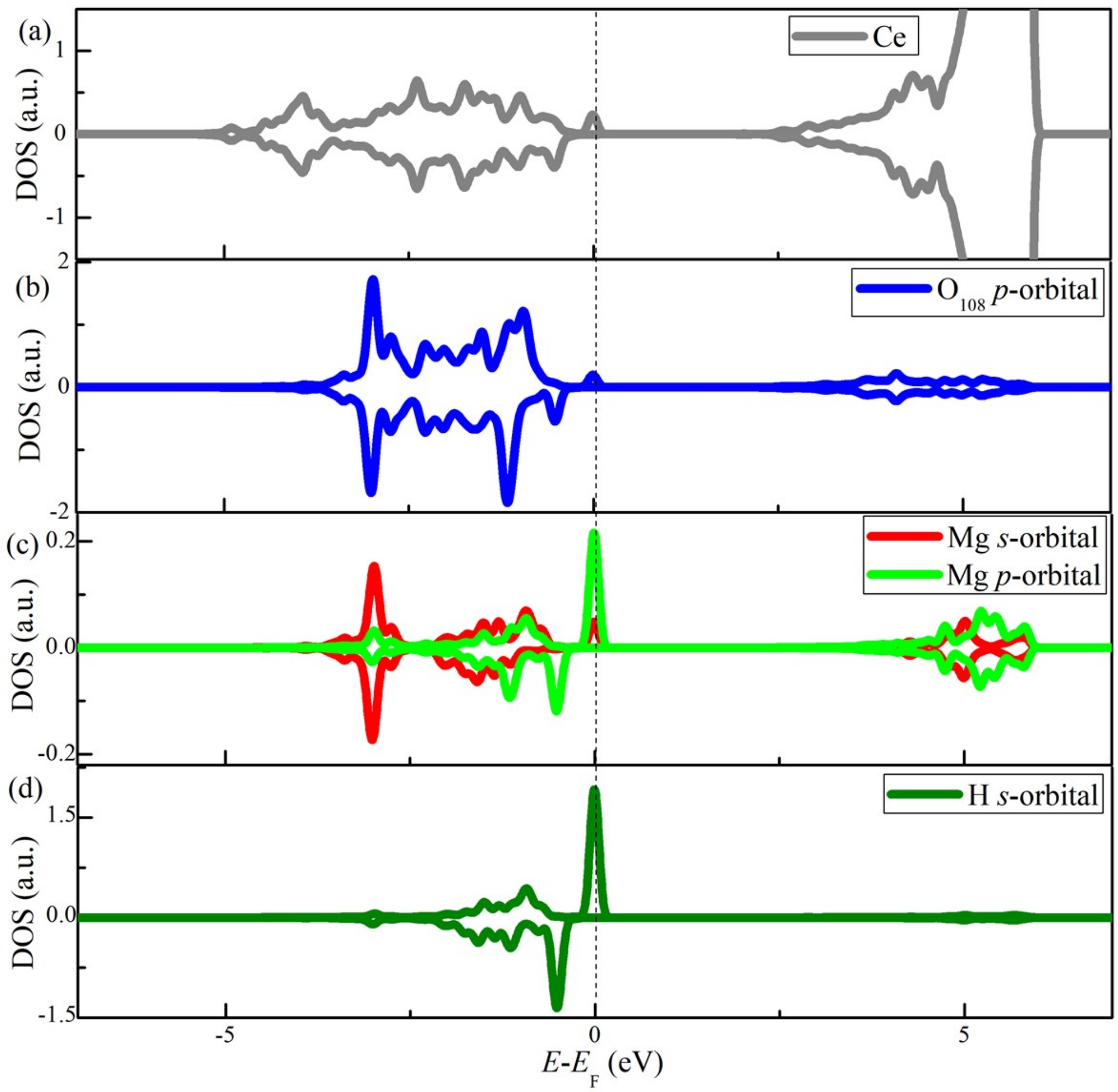

Fig. S4 PDOS distribution of MgH_2 on Ce-TiO_2 surface for (a) Ce impurity, (b) O *p*-orbitals, (c) Mg *s*- and *p*-orbitals, and (d) H *s*-orbital

S4. Structural evolution during H_2 desorption on Ce-doped TiO_2 surfaces

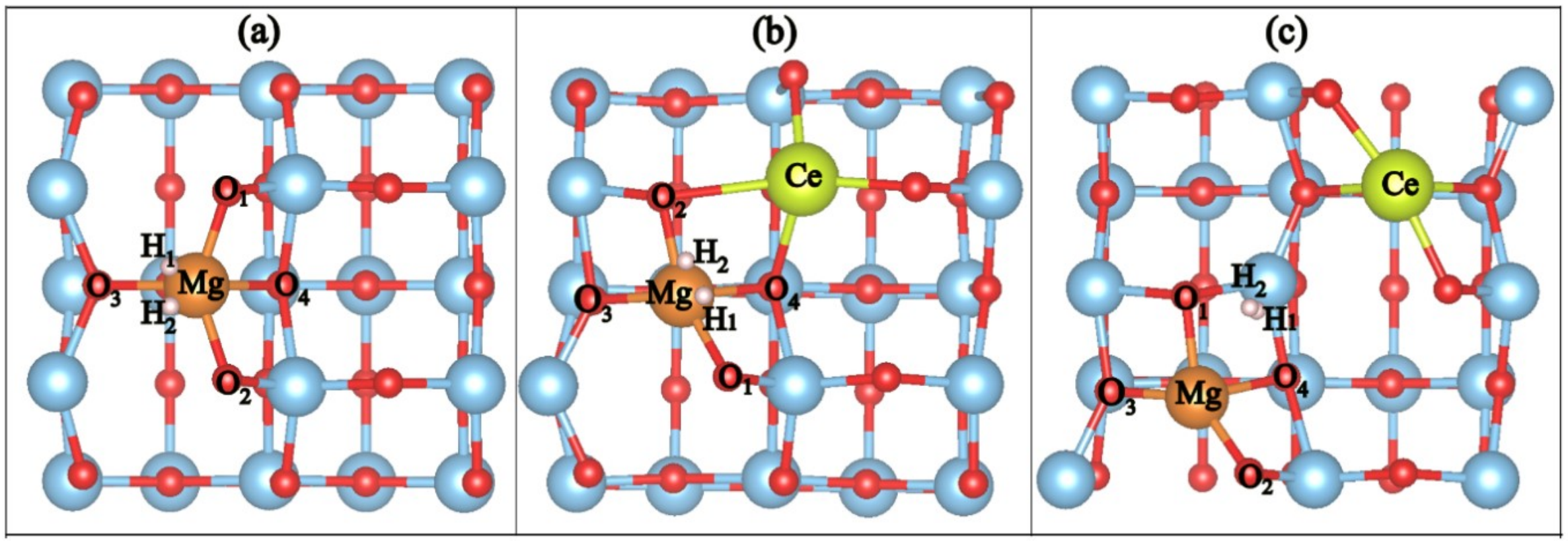

Fig. S5 Final structures after H_2 desorption on different surfaces. (a) Pristine surface; (b) Substitutional Ce-doped surface; (c) Interstitial Ce-doped surface.

As shown in Fig. S5, for all three surfaces, the H atoms desorb to form H_2 molecules residing above the surface, while the Mg ions are stabilized in tetrahedral coordination with four surface O atoms. The changes in Mg-H and Mg-O bond lengths from the initial to the final states are listed in Table S2.

Table S2 Structural evolution during H_2 desorption on pure/Ce-doped TiO_2 surfaces

**MgH_2@TiO_2**								
d_{bond} (Å)	00	01	02	03	04	05	06	07
$\boldsymbol{d}_{\mathbf{Mg\text{-}H1}}$	1.76	1.77	1.76	1.75	1.80	2.49	2.78	2.47
$\boldsymbol{d}_{\mathbf{Mg\text{-}H2}}$	1.76	1.77	1.76	1.85	2.11	2.55	2.68	2.47
$\boldsymbol{d}_{\mathbf{Mg\text{-}O1}}$	1.97	1.94	1.95	1.89	1.88	1.86	1.92	1.92
$\boldsymbol{d}_{\mathbf{Mg\text{-}O2}}$	4.95	4.94	4.02	3.09	2.11	1.87	1.92	1.92
$\boldsymbol{d}_{\mathbf{Mg\text{-}O3}}$	5.23	4.84	4.45	3.92	3.51	2.38	2.03	2.03
$\boldsymbol{d}_{\mathbf{Mg\text{-}O4}}$	4.41	4.25	4.09	3.70	3.26	2.76	2.13	2.11
**MgH_2@ substitutional Ce-doped TiO_2**								
d_{bond} (Å)	00	01	02	03	04	05	06	07
$\boldsymbol{d}_{\mathbf{Mg\text{-}H1}}$	1.78	1.88	2.64	3.26	2.31	2.68	2.88	2.68
$\boldsymbol{d}_{\mathbf{Mg\text{-}H2}}$	1.80	1.76	1.72	1.80	1.83	2.48	3.00	2.72
$\boldsymbol{d}_{\mathbf{Mg\text{-}O1}}$	1.91	1.86	1.84	1.92	1.92	1.88	1.94	1.94
$\boldsymbol{d}_{\mathbf{Mg\text{-}O2}}$	4.22	4.17	2.89	2.00	1.96	1.89	1.96	1.96
$\boldsymbol{d}_{\mathbf{Mg\text{-}O3}}$	5.17	5.12	4.08	3.32	3.21	2.34	1.98	1.97
$\boldsymbol{d}_{\mathbf{Mg\text{-}O4}}$	3.81	3.74	3.34	3.01	3.04	2.58	2.06	2.05

MgH₂@ interstitial Ce-doped TiO₂								
d_{bond} (Å)	00	01	02	03	04	05	06	07
$\boldsymbol{d}_{\textbf{Mg-H1}}$	1.96	1.96	2.47	2.62	2.56	3.20	3.65	4.66
$\boldsymbol{d}_{\textbf{Mg-H2}}$	1.70	1.70	1.74	1.72	2.41	3.58	4.17	5.32
$\boldsymbol{d}_{\textbf{Mg-O1}}$	1.92	1.93	2.09	2.19	1.97	2.03	2.02	2.03
$\boldsymbol{d}_{\textbf{Mg-O2}}$	3.81	3.81	2.12	1.94	1.87	1.91	1.91	1.91
$\boldsymbol{d}_{\textbf{Mg-O3}}$	4.03	4.03	3.42	3.46	2.43	1.99	1.99	1.99
$\boldsymbol{d}_{\textbf{Mg-O4}}$	3.73	3.72	3.12	2.62	2.19	2.09	2.09	2.10

S5. Spin distribution of RE interstitially doped TiO_2

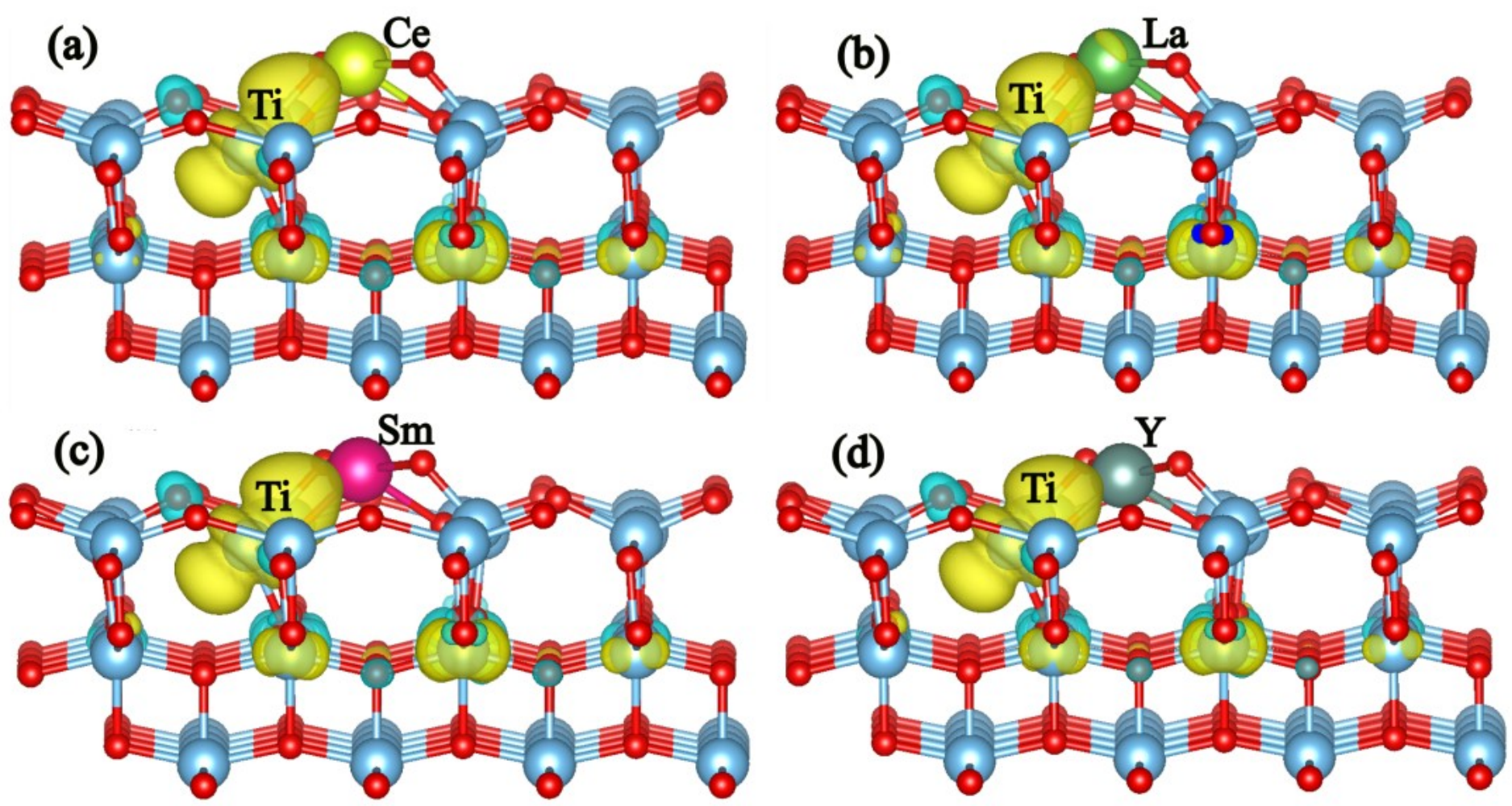

Fig. S6 Spin distribution of interstitial (a) Ce-doped, (b) La-doped, (c) Sm-doped, and (d) Y-doped TiO_2 structures. Isosurface level = $\pm 0.001 e/bohr^3$.

Substitution of a surface Ti atom by a rare-earth (RE) element yields a non-magnetic configuration, consistent with the pristine TiO_2 surface. In contrast, interstitial RE doping in the surface layer distorts the local structure and modifies the coordination environment, inducing a finite net magnetic moment of 0.83-0.85 μB that is almost entirely localized on the Ti atom neighboring the RE dopant (as shown in Fig. S6).

S6. Bader charge analysis of the MgH_2 adsorption systems

Bader charge analysis quantitatively reveals the following charge transfer trends, see the details in Table. S3. In all systems, Mg consistently loses electrons ($\Delta Q_{Mg} < 0$) while the surface O gains electrons ($\Delta Q_O > 0$), confirming Mg→O as the primary charge transfer channel. On the pristine TiO_2 surface, Mg loses ~0.12 e^- and O gains ~0.18 e^-. For substitutional Ce/La/Sm doping, Mg loss increases to ~0.18 e^- and O gains ~0.33 e^- accompanied by slight electron loss on RE (~0.03 e^-). These quantitative results corroborate the RE–H coupling channel observed in the charge density difference analysis, indicating an additional RE→H2 charge transfer pathway beyond the primary Mg–O channel. In sharp contrast, for substitutional Y doping, Mg loss increases dramatically to 1.61 e^-, while the surface O gain reaches 0.42 e^-, with no charge transfer observed on Y ($\Delta Q_Y = 0$), indicating that Y promotes dissociative adsorption through enhanced Mg-O charge redistribution rather than direct charge transfer. For interstitial doping, similar charge transfer behavior is observed: Mg loses 0.16-0.28 e^-, O gains 0.17-0.19 e^-, and all RE dopants show slight electron loss (~0.04 e^-), further confirming the universality of the RE-H2 charge transfer pathway.

Table S3 Bader charge variations for MgH_2 adsorption on pristine/RE-doped TiO_2 surfaces

**Surface**	**Doping Mode**	ΔQ_{Mg}	ΔQ_{H1}	ΔQ_{H2}	ΔQ_O	ΔQ_{Ti}	ΔQ_{RE}
**Pristine**	——	-0.12	0.02	0.02	0.18	-0.05	——
**Ce-TiO_2**	Substitutional	-0.18	-0.16	0.04	0.33	-0.04	-0.03
**La-TiO_2**	Substitutional	-0.18	-0.16	0.04	0.33	-0.04	-0.03
**Sm-TiO_2**	Substitutional	-0.18	-0.16	0.04	0.33	-0.04	-0.03
**Y-TiO_2**	Substitutional	-1.61	-0.06	-0.02	0.42	0.12	0
**Ce-TiO_2**	Interstitial	-0.16	-0.03	0.03	0.19	-0.03	-0.04
**La-TiO_2**	Interstitial	-0.16	-0.03	0.03	0.19	-0.03	-0.04
**Sm-TiO_2**	Interstitial	-0.28	-0.03	0.05	0.18	-0.03	-0.04
**Y-TiO_2**	Interstitial	-0.26	-0.02	0.05	0.17	-0.04	-0.04

S7. CI-NEB result for MgH_2 dehydrogenation on Y-doped TiO_2 surface

We performed additional CI-NEB calculations for Y interstitial doping. Notably, in contrast to substitutional Y doping, the interstitial Y doping configuration preserves semi-dissociative adsorption of MgH_2, thereby allowing conventional CI-NEB analysis. The potential energy profile along this reaction pathway exhibits a largely monotonic decreasing trend after adsorption (see Fig. S7), suggesting two possible scenarios: 1) the dehydrogenation process proceeds spontaneously to the final stable state without overcoming a significant energy barrier; or 2) the process involves an extremely low barrier transition state that falls below the detection limit of the present CI-NEB calculations. Although our current calculations cannot unequivocally distinguish between these two scenarios, both interpretations converge to the same conclusion—that Y doping effectively promotes the dehydrogenation kinetics of MgH_2, confirming its beneficial catalytic role.

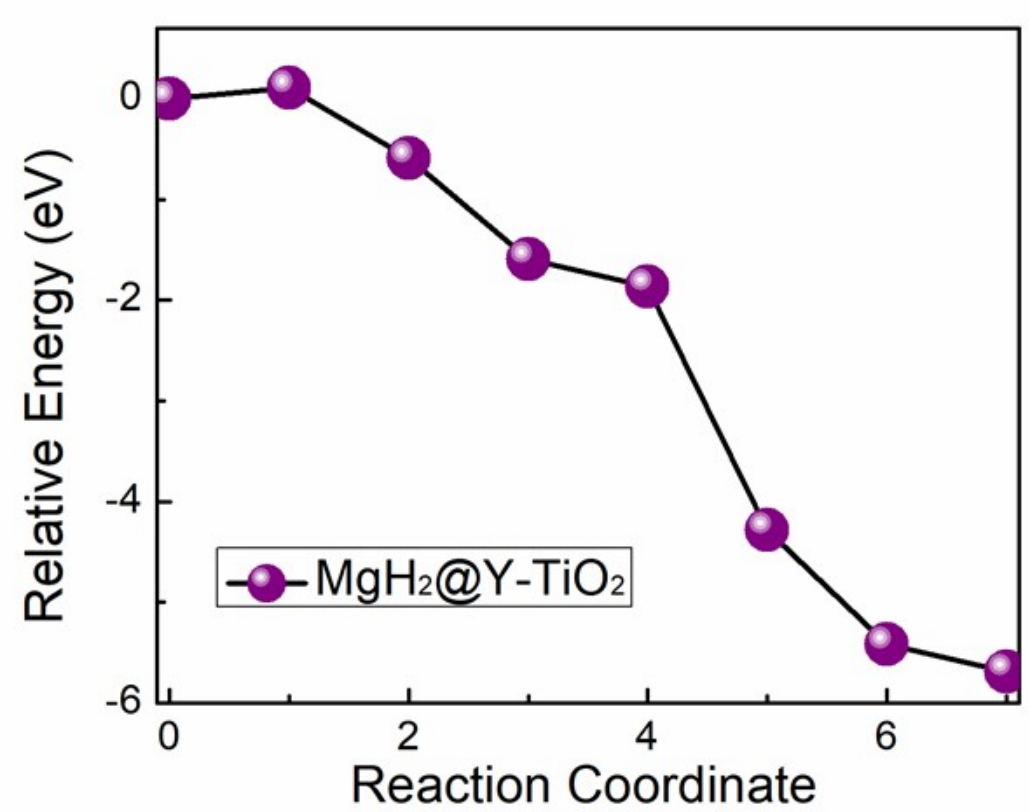

Fig. S7 The relative energy profiles for the dissociation process on Y-interstitial doped TiO_2
